# Changes in Anxiety, Depression, and Stress in 1 Week and 1 Month Later After the Wuhan Shutdown Against the COVID-19 Epidemic

**DOI:** 10.1017/dmp.2021.20

**Published:** 2021-01-21

**Authors:** Qi Chen, Mengying Li, Yueqing Wang, Ling Zhang, Xiaodong Tan

**Affiliations:** 1 Department of Occupational and Environmental Health, School of Health Sciences, Wuhan University; Wuhan, China; 2 Department of Epidemiology and Biostatistics, School of Public Health, Peking University, Beijing, China

**Keywords:** COVID-19, shutdown, anxiety, depression, stress

## Abstract

**Objective:**

The aim of this study was to investigate the changes in Chinese residents’ psychological state and its influencing factors after the Wuhan shutdown on January 23, 2020.

**Methods:**

Two surveys were conducted on February 1-5 and February 20-24, separately, using an online self-administrated questionnaire among 3145 and 3814 participants, respectively. Subjective indicators of daily-life changes include level of attention, risk of infection, impact of daily life, self-perceived health status, and mental health help-seeking. Individual scores on changes in anxiety, depression, and stress were generated by 6-item, 4-item, and 3-item questions. A multivariate regression model was fitted in each survey, separately and combined.

**Results:**

A total of 6959 residents participated in the study, with 32.78% male and 67.22% female, people living in Wuhan and other cities in Hubei Province accounted for 25.22% and 18.85%, respectively. One week after the Wuhan shutdown, their anxiety, depression, and stress had all increased. Compared with the first survey, the changes in the scores of anxiety, depression, and stress in the second survey were decreased (β = −1.220, −0.798, and −0.623, all *P* < 0.001). The level of attention, risk of infection, and self-perceived health status tended to be positively associated with the changes in the scores of anxiety, depression, and stress.

**Conclusions:**

The study showed that the lives and psychological conditions of residents had undergone negative changes after the Wuhan shutdown, but the measures taken during this period were effective. These results may provide guidance for public health policies in other countries and regions.

A series of pneumonia cases of unknown origin occurred in Wuhan, China on 31 December, 2019.^1^ On January 31, 2020, the coronavirus disease 2019 (COVID-19) outbreak, with its epicenter in Wuhan, was declared a public health emergency of international concern by the World Health Organization (WHO).^2^ Considering that the disease is highly contagious and, to prevent the further spread of COVID-19 from its source, all transport to and from Wuhan city was prohibited from 10:00 am on January 23, 2020, followed by the entire Hubei Province a day later, which has been proved to be effective and was estimated to have stopped at least 700,000 cases of COVID-19.^3^ Also, in the absence of vaccination and treatment, other actions were taken in the whole country to prevent further spread, including stay-at-home order, social distancing, facemask wearing, temperature check, and restrictions on going out per family per household. Previous evidence showed that quarantine and isolation of patients led to widespread fear and panic, resulting in negative psychological reactions, including adjustment disorder and depression.^4-8^

Meanwhile, domestic and foreign media were scrambling to report the COVID-19 epidemic, which may trigger a psychological crisis when most residents were confined at home. The high prevalence of mental health problems was positively related to the frequent social media exposure during the COVID-19 outbreak.^9^ People who search online or use social media to post information about the epidemic were more likely to have psychological distress.^10^ One recent study has noted an increase of psychological problems during this epidemic, including anxiety, depression, and stress.^11^ Two studies emphasized that we should pay attention to the mental health of specific groups, such as quarantined children, older adults, patients, and medical staff.^12,13^ It turns out that pandemic disaster and subsequent disease control measures caused psychological harm to these groups.^14-16^ However, no data are available on the public psychological changes after the Wuhan shutdown in fighting against COVID-19.

In the present study, we conducted 2 surveys to investigate the residents’ changes in life and psychological conditions in 1 wk and 1 mo later, respectively, after the Wuhan shutdown. The 5 indicators used to measure these changes include level of attention, self-assessed infection risk, impact of daily life, self-perceived health status, and mental health help-seeking. We are primarily focused on comparing the changes of residents’ psychological status at 2 different time points, which can reflect the effectiveness of government measures during this period. Second, we aim to identify factors affecting the psychological status of residents, so that relevant personnel can pay more attention to people with worse psychological conditions during the epidemic, and provide them with better psychological support.

## Methods

### Study Participants

In 2020, We conducted 2 surveys 1 wk (February 1 to 5) and 1 mo (February 20 to 24) after the shutdown of Wuhan (January 23) and Hubei Province (January 25) against the COVID-19 spread. Participants in this study were recruited online. Because all means of transportation were forbidden during the survey period, no face-to-face communication could be conducted, so all our questionnaires were conducted on social networks. We disseminated a self-reported questionnaire to the general public in China, by means of WeChat. We received 3284 and 4071 anonymous questionnaires in 2 investigations, respectively, covering 33 Chinese provinces and autonomous regions except for Taiwan.

Inclusion criteria: (1) male or female, ages 15-85 y; (2) participants must have the capacity to understand the study and provide informed consent; and (3) participants must be fluent in Chinese.

Exclusion criteria: (1) serious neurological (specific or focal) disorders preventing full participation in the protocol; (2) illogical responses in the questionnaire (eg, selecting the same option consecutively, the results of similar choices vary widely).

After eliminating the invalid samples, 3145 (95.77%) and 3814 (93.69%) valid questionnaires were finally obtained. This study was approved by the research ethics committees of Wuhan University. All participants provided informed consent. Based on the investigation of the psychological state after the disaster in China and compiled after the discussion of experts, the self-administrated questionnaires were mainly divided into 3 parts, including sociodemographic characteristics, changes in psychological status, and subjective indicators of changes in daily life.

### Changes in Psychological Status

Changes of psychological status: In the study, 11 feeling items were used to measure the change of psychologic status, including sorrow, fear, tired, irritability, loneliness, sleep condition, self-perceived uselessness, irritability and loneliness, weight, appetite, chest tightness, disturbed, and muscle ache. We rated these items in a 5-point response format: −2 = significantly decreased, −1 = decreased, 0 = unchanged, 1 = increased, or 2 = significantly increased. Literature review and experts interview methods were used to construct the index system to sort the items and calculating the scores and to analyze the changes in the residents’ psychological status. According to the literature,^17-19^ the 11 feeling items in the homemade questionnaires were classified into 3 categories: anxiety, depression, and stress.

The total scores were calculated by simple addition based on the extent of the feeling. A negative score indicated that the negative emotions of the participants decreased compared with the previous week; otherwise, a positive score indicated that the negative emotions increased. The higher the score, the worse the psychological condition was. The reliability of the questionnaire was checked using Cronbach’s alpha, and the reliability coefficients of anxiety, depression, and stress were 0.815, 0.695, 0.560, respectively.

### Subjective Indicators of Changes in Daily Life

The status of daily life of residents after the Wuhan shutdown is composed of level of attention, self-assessed infection risk, impact of daily life, self-perceived health status, and mental health help-seeking. The first 3 items were rated as −2 = significantly decrease, −1 = decreased, 0 = unchanged, 1 = increased, or 2 = significantly increased; while the self-perceived health status was rated as 2 = fairly healthy, 1 = healthy, 0 = general, −1 = unhealthy but live independently, −2 = unable to live independently. The item of mental health help-seeking was related as 2 = found and tried, 1 = found but not tried, 0 = not found yet, −1 = not looked for, −2 = no need to adjust.

### Covariates

Gender (1 = male, 0 = female), age(1 = ≤18, 2 = 19-29, 3 = 30-39, 4 = 40-49, 5 = 50-59, 6 = ≥60,) education (1 = elementary school or below, 2 = middle school, 3 = high school, 4 = college, 5 = masters degree and above), area (1 = city, 2 = rural), current residence (1 = Wuhan, Hubei, 2 = other cities in Hubei, 3 = other provinces and cities, 4 = overseas), marital status (1 = single, 2 = married, 3 = separated/divorced, 4 = other), occupation (0 = nonmedical staff, 1 = medical staff), monthly income (Yuan) (1 ≤ 2000, 2 = 2000-5000, 3 = 5001-10,000, 4 = 10,001-15,000, 5 ≥ 15,001), number of cohabitants (1 = 0, 2 = 1, 3 = 2-3, 4 = ≥ 4), quarantine or not (0 = no, 1 = yes), confirmed cases in personal network (0 = no, 1 = yes).

### Statistical Analysis

Data were double-entered and cross-checked using Excel version 2019 (Microsoft Corp.; Redmond, WA), R3.6.2 was used for data cleaning and drawing the original distribution map of the 3 psychological feelings, Statistical Package for the Social Sciences (SPSS 25.0; SPSS Inc., Chicago, IL) was used to conduct corresponding statistical analysis, and a 2-sided *P* value less that 0.05 was considered statistically significant.

To identify the determinants of participants’ psychological feelings, we first examined the effects of their characteristics on changes of anxiety, depression, and stress scores with 1-way analysis of variance (ANOVA) or the nonparametric Kruskall-Wallis test for categorical variables, depending on the distribution of the variables. The statistically significant variables were then allowed to enter into the multiple linear regression model, and dummy variables were created when appropriate. Multivariate regression models were fitted to investigate the associations between the changes of daily life and changes in psychological scores, with adjustment of age, gender, education, marital status, occupation, monthly income, quarantine, and confirmed infected in personal network. Finally, the various models were tested for the presence of significant interaction between the first and second survey.

## Results

A total of 6959 residents participated in the study, 32.78% male and 67.22% female (Table 1). Participants aged 18 y and younger, 19-29 y old, 30-39 y old, 40-49 y old, 50-59 y old, 60 y old and older accounted for 2.24%, 27.59%, 29.21%, 26.25%, 12.73%, 1.97%, respectively. A total of 78.27% of study participants were urban residents. Among the participants, people living in Wuhan and other cities in Hubei Province accounted for 25.22% and 18.85%, respectively. Residents in and outside Hubei Province each accounted for approximately half. The data showed that most of the participants had university degrees (64.20%) or above (17.98%), more than half (67.28%) of the subjects were married. The proportion of medical staff was 21.87%. At the time of the data collection, 1868 (28.84%) people were in quarantine and 1192 (17.13%) said that people they knew were diagnosed with COVID-19. Results of univariable analyses were shown in Supplementary Tables 1 and 2.

**Table 1.** Sociodemographic characteristics of participants (*N* = 6959)

Variable	Total (*n* = 6959)	1^st^ Survey (*n* = 3145)	2^nd^ Survey (*n* = 3814)	*P*
Sex				0.002
Male	2281 (32.78)	969 (30.81)	1312 (34.40)	
Female	4678 (67.22)	2176 (69.19)	2502 (65.60)	
Age (y)				<0.001
≤18	156 (2.24)	13 (0.41)	143 (3.75)	
19-29	1920 (27.59)	911 (28.97)	1009 (26.46)	
30-39	2033 (29.21)	1033 (32.85)	1000 (26.22)	
40-49	1827 (26.25)	835 (26.55)	992 (26.01)	
50-59	886 (12.73)	313 (9.95)	573 (15.02)	
≥60	137 (1.97)	40 (1.27)	97 (2.54)	
Area				0.199
City	5447 (78.27)	2484 (78.98)	2963 (77.69)	
Rural	1512 (21.73)	661 (21.02)	851 (22.31)	
Current residence				<0.001
Wuhan, Hubei	1755 (25.22)	560 (17.81)	1195 (31.33)	
Other cities in Hubei	1312 (18.85)	389 (12.37)	923 (24.20)	
Other provinces and cities	3875 (55.68)	2191 (69.67)	1684 (44.15)	
Overseas	17 (2.45)	5 (0.15)	12 (0.31)	
Education				<0.001
Elementary school or below	20 (0.29)	7 (0.22)	13 (0.34)	
Middle school	264 (3.79)	147 (4.67)	117 (3.07)	
High school	956 (13.74)	467 (14.85)	489 (12.82)	
College	4468 (64.20)	2050 (65.18)	2418 (63.40)	
Master degree and above	1251 (17.98)	474 (15.07)	777 (20.37)	
Marital status				0.061
Single	1936 (27.82)	832 (26.45)	1104 (28.95)	
Married	4682 (67.28)	2145 (68.20)	2537 (66.52)	
Separation/divorced	291 (4.18)	141 (4.48)	150 (3.93)	
Other	50 (0.72)	27 (0.86)	23 (0.60)	
Occupation				<0.001
Medical staff	1522 (21.87)	507(16.12)	1015(26.61)	
Non-medical staff	5437 (78.13)	2638(83.88)	2799(73.39)	
Monthly income(Yuan)				<0.001
<2000	1194 (17.16)	414 (13.16)	780 (20.45)	
2000-5000	2185 (31.40)	1040 (33.07)	1145 (30.02)	
5001-10000	1978 (28.42)	947 (30.11)	1031 (27.03)	
10001-15000	824 (11.84)	386 (12.27)	438 (11.48)	
>15000	778 (11.18)	358 (11.38)	420 (11.01)	
Number of cohabitants				<0.001
0	269 (3.87)	50 (1.59)	219 (5.74)	
1	696 (10.00)	239 (7.60)	457 (11.98)	
2-3	3843 (55.22)	1917 (60.95)	1926 (50.50)	
≥4	2151 (30.91)	939 (29.86)	1212 (31.78)	
Quarantine or not				<0.001
Yes	1868 (26.84)	1436 (45.66)	432 (11.33)	
No	5091 (73.16)	1709 (54.34)	3382 (88.67)	
Confirmed infected in personal network				<0.001
Yes	1192 (17.13)	472 (15.01)	720 (18.88)	
No	5767 (82.87)	2673 (84.99)	3094 (81.12)	

**Figure 1.**
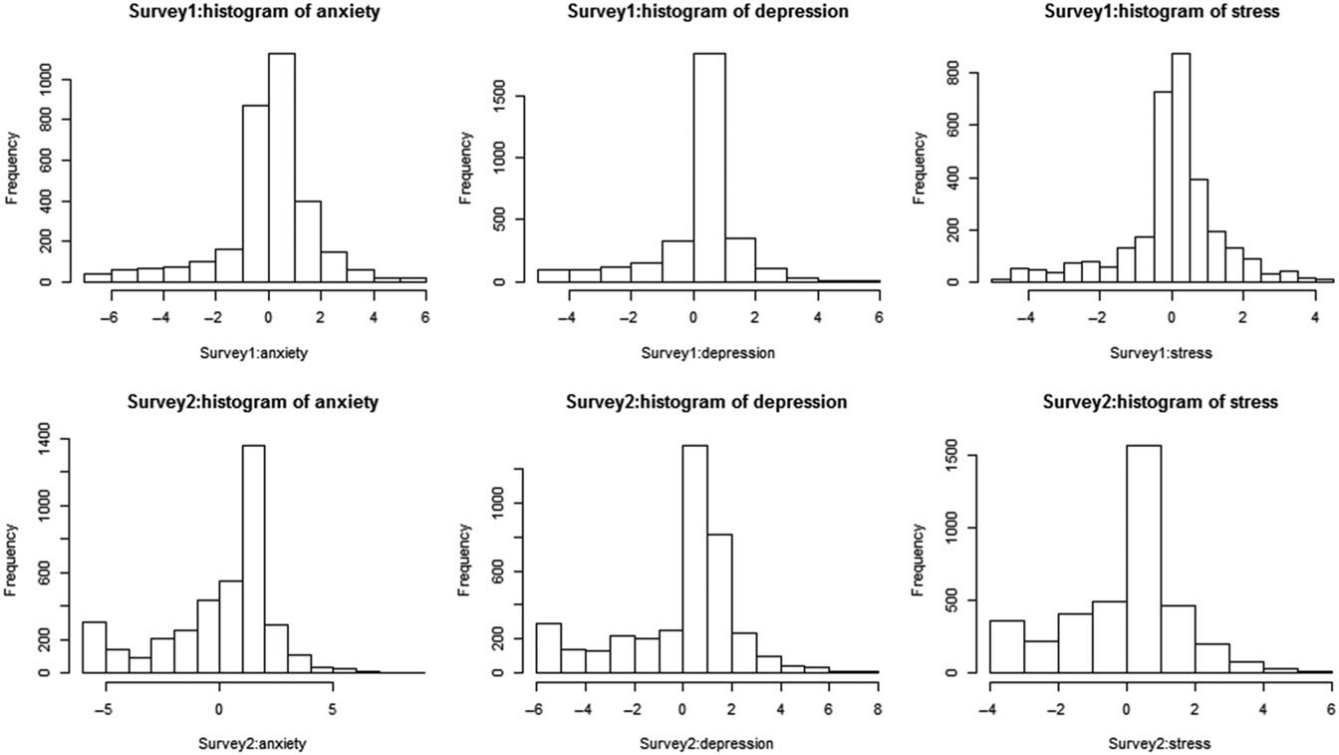
Distribution of changes in scores of depression, anxiety, and stress scores in 2 surveys.

In Figure 1, the changes in the scores of negative emotions show a normal distribution. In the first survey, 1 wk after the Wuhan shutdown, the scores of people’s anxiety (0.97 ± 3.40), depression (0.58 ± 1.92), and stress (0.39 ± 1.71) showed a significant increase, while from first to second assessment, the results showed that the scores of people’s anxiety (-1.99 ± 3.88), depression (-0.99 ± 2.09) and stress (-0.88 ± 1.89) decreased.

**Figure 2.**
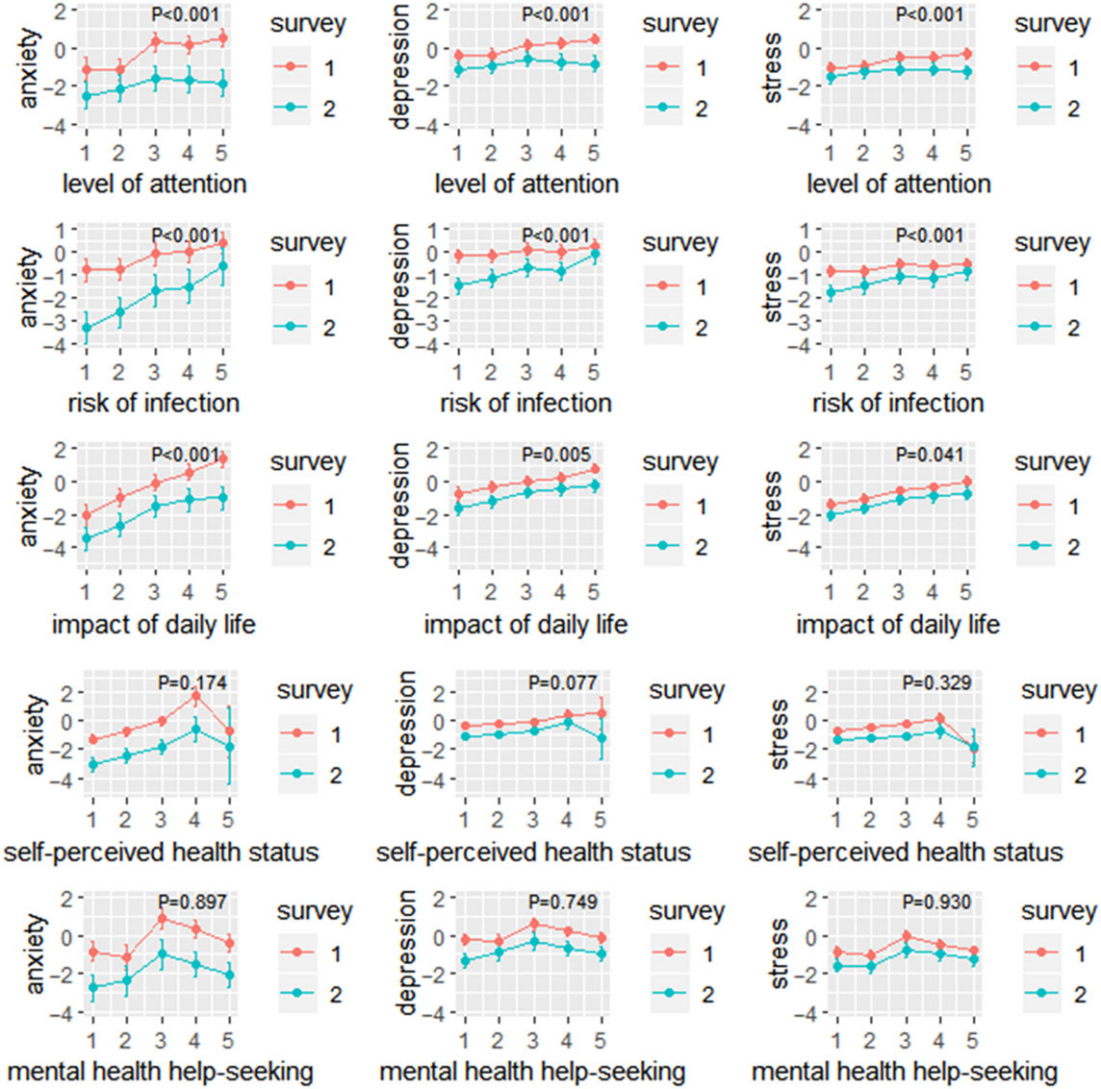
The adjusted means of changes in anxiety, depression, and stress score according to 5 subjective indicators of changes of daily life in the 2 surveys. The *P*-value in the figure represents the result of the interaction between the according subjective indicator (including level of attention, risk of infection, impact of daily life, self-perceived health status, and mental health help-seeking) and surveys (survey 1 and 2) on the changes of 3 psychological scores (including anxiety, depression, and stress).

Figure 2 show that the changes in the scores of anxiety, depression, and stress increased to some extent at different periods. The scores were lower in the second survey than in the first survey, indicating that people’s psychological conditions were getting better. The first 3 self-perception factors, namely the level of attention, the risk of infection, and the impact of daily life, were positively correlated with the scores of the 3 negative emotions. As participants’ self-perceived health status and their need for seeking mental health help decreased, the scores of the 3 psychological conditions showed a trend of increasing first and then decreasing. Compared with other participants, those who chose “unhealthy but able to take care of themselves” had the highest negative emotion scores in their self-perceived health status. Participants who tried to find mental health help but did not find had the highest negative emotion scores.

The interaction effect for the survey of time showed that the level of attention had effects on the scores of anxiety (*β*-coefficient = −0.427; SE = 0.088; *P* < 0.001), depression (*β*-coefficient = −0.241; SE = 0.048; *P* < 0.001), and stress (*β*-coefficient = −0.195; SE = 0.043; *P* < 0.001) in the 2 surveys; the risk of infection had different effects on the scores of anxiety (*β*-coefficient = 0.598; SE = 0.075; *P* < 0.001), depression (*β*-coefficient = 0.318; SE = 0.042; *P* < 0.001), and stress (*β*-coefficient = 0.295; SE = 0.038; *P* < 0.001) in the 2 surveys. An interaction model included survey of time × impact of daily life, which was significant on the scores of anxiety (*β*-coefficient = −0.230; SE = 0.091; *P* < 0.001), depression (*β*-coefficient = −0.082; SE = 0.051; *P* = 0.005), and stress (*β*-coefficient = −0.045; SE = 0.045; *P* = 0.041).

In multiple linear regression of residents’ scores of anxiety, depression, and stress in 2 surveys (Table 2), age and gender were included in Model 1 as control variables. The control variables of Model 2 were age, gender, education, marital status, occupation, and monthly income. Model 3 controlled all covariates in the surveys, that is, quarantine and confirmed infected in personal network were added on the basis of Model 2. The results of the 3 models showed that the level of attention was positively correlated with the changes in the scores of aniexty (*β*-coefficient = 0.115; *P* < 0.001), depression (*β*-coefficient = 0.121; *P* < 0.001), and stress (*β*-coefficient = 0.100; *P* < 0.001).

**Table 2.** Multivariable linear regression of residents’ scores of anxiety, depression, and stress in 2 surveys (*n* = 6959)

	Anxiety			Depression			Stress		
Independent Variable	Model 1	Model 2	Model 3	Model 1	Model 2	Model 3	Model 1	Model 2	Model 3
Level of attention	0.118* (0.034)	0.123 * (0.035)	0.115* (0.035)	0.121* (0.019)	0.128* (0.019)	0.121* (0.020)	0.097* (0.017)	0.104* (0.017)	0.100* (0.018)
Risk of infection	0.192* (0.036)	0.189* (0.036)	0.183* (0.036)	0.154* (0.020)	0.152* (0.020)	0.147* (0.020)	0.144* (0.018)	0.140* (0.018)	0.137* (0.018)
Impact of daily life	0.240* (0.043)	0.238* (0.043)	0.231* (0.044)	0.239* (0.024)	0.237* (0.024)	0.231* (0.025)	0.234* (0.022)	0.230* (0.022)	0.227* (0.022)
Self-perceived health status	0.149* (0.063)	0.152* (0.063)	0.149* (0.063)	0.083* (0.035)	0.086* (0.035)	0.084* (0.036)	0.108* (0.032)	0.111* (0.032)	0.110* (0.032)
Mental health help-seeking	0.020 (0.041)	0.019 (0.041)	0.019 (0.041)	0.014 (0.023)	0.013 (0.023)	0.012 (0.023)	0.019 (0.021)	0.017 (0.021)	0.017 (0.021)

Note: Model 1, adjusted for age and sex; Model 2, adjusted for age, sex, education, marital status, occupation, and monthly income; Model 3, adjusted for age, sex, education, marital status, occupation, monthly income, quarantine, and confirmed infected in the personal network.

*:*P* < 0.001.

Respondents who thought they were more likely to be infected had higher scores of anxiety (*β*-coefficient = 0.183; *P* < 0.001), depression (*β*-coefficient = 0.147; *P* < 0.001), and stress (*β*-coefficient = 0.138; *P* < 0.001). People whose lives were affected more by the shutdown scored higher on the changes in the scores of anxiety (*β*-coefficient = 0.231; *P* < 0.001), depression (*β*-coefficient = 0.231; *P* < 0.001), and stress (*β*-coefficient = 0.227; *P* < 0.001). Similarly, the worse the self-perceived health status, the higher the scores of anxiety (*β*-coefficient = 0.149; *P* < 0.001), depression (*β*-coefficient = 0.084; *P* < 0.001), and stress (*β*-coefficient = 0.110; *P* < 0.001).

## Discussion

This study has demonstrated that the COVID-19 outbreak increased the negative sentiment of the Chinese public a week after the Wuhan shutdown. To the best of our knowledge, the present investigation is the first study to characterize the changes in psychological status in 2 different periods after the Wuhan shutdown.

In both surveys about negative emotions conducted at 1 wk and 1 mo after the Wuhan shutdown, the changes in the scores of anxiety, depression, and stress generally increased at 1 wk after the Wuhan shutdown on January 23 (indicating worsening phychological conditions), while showed a slight decline at the second investigation around February 20 (indicating improved phychological conditions). These data provide evidence that the outbreak of infectious diseases may have profound psychological effects.^20^ It means that the measures taken by Wuhan government during the 2 surveys to prevent COVID-19 facilitated the regulation of psychological state of respondents over time. It may be that when Wuhan was just shut, most of the participants were in a relatively unfamiliar stage for COVID-19, and felt puzzled and panic about the route of infection. In mid-February, with the opening of various information channels, the continuous support of the national medical team,^21^ the rise of production capacity of COVID-19 diagnostic kits and various medical supplies, including mobile hospitals for cases of COVID-19 with mild symptoms and “Leishenshan” and “Huoshenshan” hospitals to treat cases of COVID-19 with some emergency warning signs being built,^22-24^ the distress of negative emotions reduced.

In addition, the upturn of the epidemic has also led to a shift in emotion. The reported daily incidence of confirmed cases peaked in Hubei province on February 4, and in all other provinces on January 31. Since February 18, the daily number of cases recovered was greater than the daily number of new confirmed case for the first time. During the 2 investigations, medical conditions and resources had been greatly improved, and “4 types of personnel” were quarantined and treated in a concentrated manner. At the same time, the government had continuously upgraded the management and control measures for communities and ordinary citizens, adopting closed communities and centralized distribution of living materials to further reduce the risk of community infection. All these measures had been proved to be effective in preventing the spread of the epidemic,^25^ and our results also reflected from the side that these had also reduced the anxiety, depression, and stress of residents.

The results extend previous findings and highlight the influence of the level of attention, impact of daily life, self-assessed infection risk, self-perceived health status to negative psychological status. Attention to COVID-19, risk of infection and self-perceived health status were associated with higher levels of anxiety, depression, and stress. Respondents who believed that their lives were more severely affected by the COVID-19 exhibited more obvious anxiety, depression, and stress than others.^26^ Specifically, we found that urban residents were more likely to report anxiety, depression, and stress than rural counterparts. This effect may be due to the dense population of urban areas. Well-planned efficient public transportation systems can facilitate residents’ travel.^27^ However, the shutdown suspended all operations of public transportation, subways and other means of transportation, affecting essential travel for city residents, especially those who had to go to work during the outbreak. A more important reason is that, due to the high density of the urban population and the greater mobility of people than rural areas, the risk of disease infection is greater. High population density increases people’s exposure to infectious diseases,^28^ which may lead to increased negative emotions among urban residents. The disruption of daily life and the limitations of entertainment or recreation made it more difficult for urban dwellers to release their excess inner pressure than rural residents. The result gives an indication that day to day life for the residents will be improved regarding mental health.^29^

Our study also showed that, the greater the level of attention to COVID-19, the greater the negative emotions, which is in agreement with previous research.^30^ As a result of strict prevention and control measures, the general awareness and vigilance of residents have increased.^31,32^ Respondents were those who had mobile phones with access to WeChat, and decided to participate in the survey, which means that most people got information about COVID-19 mainly through the mass media. However, because our investigations were conducted in the early stage of shutdown, everyone had inadequate understanding of the actual situation of COVID-19, so discrimination and prejudice driven by fear or misinformation had been flowing globally.^26^ Because people cannot differentiate true and false news, the more attention to COVID-19, the more unclear information may be received, which negatively affects respondents’ psychological status. As a result, the emotion of this group is more vulnerable to the unjustified information that has been rampant during the COVID-19 outbreak.

In addition, the elevation of the changes in the scores of anxiety, depression, and stress were positively associated with the increase of self-assessed infection risk, and with the decrease of self-perceived health status. Respondents received signals from the surrounding environment and were supposed to make corresponding assessment of their risk of infection. This is consistent with previous research^33^ showing that negative emotions, such as anxiety and loneliness, activate cognitive mechanisms that lead to poor self-assessed health and a higher likelihood of self-assessed infection. Respondents in other countries and districts may pay more attention and be more vigilant about COVID-19 due to the Wuhan shutdown. However, during the period of our surveys, the outbreaks abroad had not yet occurred, respondents who were overseas had lower ratings of infection risk, resulting in less negative emotions.^34^ Self-perceived health status provided an overall evaluation of current mental and physical health, as well as the trajectory of health.^35^ Due to the impact of the epidemic, medical resources were greatly tilted toward the prevention and treatment of COVID-19, resulting in limited medical resources for respondents with disease states. In contrast, healthy respondents had less demand for conventional medical resources, so their psychological endurance may be better.

### Limitations

The limitations of this study should not be ignored. First, data collection was completed by distributing questionnaires online. This kind of Web-based investigation has inherent flaws. During the process of data collection, sources of bias include potential selection bias of respondents, as respondents were asked if they were willing to participate in the survey, resulting in volunteer bias and may not be possible to evaluate people whose psychological conditions were severely affected by COVID-19, which means that the results cannot be truly representative of the general population. Second, the populations of the 2 surveys were different, and there were some differences in demographic characteristics. Third, although we have a sufficient number of respondents, the sampling method may have nonresponse bias by 2 surveys.^36^ Finally, the questionnaire for this study is self-designed and contains insufficient items, resulting in low reliability and validity. This is what needs to be improved in subsequent research.

## Conclusions

In conclusion, the life and psychological state of the urban population had produced negative changes after the Wuhan shutdown on January 23. However, with the gradual achievements of preventing COVID-19, the negative emotion of participants had been alleviated to some extent. During the period of shutdown, residents who were concerned about the development of the COVID-19, whose daily life was affected greatly, and who were in poor health were more likely to have negative emotions. At present, China has achieved great success in the fight against epidemics, but the epidemic situation in some parts of the world has not improved. The relevant departments need to formulate and implement actions to minimize the psychological distress of the masses to meet the needs of areas affected by COVID-19. For other affected countries, promoting the issuance of guiding regulations is of paramount significance. Currently, countries where the epidemic is still severe should implement mental health services and allocate resources to ensure that there are professionals to provide psychological guidance to individuals, thereby reducing the psychological damage that may be caused during isolation.

## Data Availability

The data that support the findings of this study are available from the corresponding author upon reasonable request.

## Supplementary material

For supplementary material accompanying this paper visit http://dx.doi.org/10.1017/dmp.2021.20. click here to view supplementary material
