# Supplementary material for: Changes in Anxiety, Depression, and Stress in 1 Week and 1 Month Later After the Wuhan Shutdown Against the COVID-19 Epidemic
Source: Disaster Med Public Health Prep. 2021 Jan 21:1–8. doi: 10.1017/dmp.2021.20 (PMC8027553; doi:10.1017/dmp.2021.20)
Supplement: Supplementary file 1 [file S1935789321000203sup001.docx]

# Appendix

**Table S1. Factors affecting anxiety, depression, and stress in the 1^st^ survey （n = 3145；X±S ;n/%）**

| **Variable** | **N** | **Anxiety** | **t/F** | ***P*** | **Depression** | **t/F** | ***P*** | **Stress** | **t/F** | ***P*** |
| --- | --- | --- | --- | --- | --- | --- | --- | --- | --- | --- |
| Gender |  |  | 1.215 | 0.270 |  | 1.316 | 0.251 |  | 2.032 | 0.154 |
| male | 969 | 1.07±3.32 |  |  | 0.64±1.98 |  |  | 0.46±1.69 |  |  |
| female | 2176 | 0.92±3.43 |  |  | 0.55±1.90 |  |  | 0.36±1.72 |  |  |
| Age |  |  | 7.479 | ﹤0.001 |  | 6.142 | ﹤0.001 |  | 6.618 | 0.001 |
| ≤18 | 13 | 1.15±2.51 |  |  | 0.08±1.85 |  |  | 0.15±1.57 |  |  |
| 19-29 | 911 | 1.32±3.10 |  |  | 0.79±1.79 |  |  | 0.57±1.61 |  |  |
| 30-39 | 1033 | 1.17±3.32 |  |  | 0.66±1.88 |  |  | 0.49±1.66 |  |  |
| 40-49 | 835 | 0.61±3.63 |  |  | 0.35±2.05 |  |  | 0.17±1.80 |  |  |
| 50-59 | 313 | 0.33±3.72 |  |  | 0.34±1.98 |  |  | 0.18±1.83 |  |  |
| ≥60 | 40 | 0.03±3.20 |  |  | 0.35±2.02 |  |  | 0.20±1.67 |  |  |
| Area |  |  | 0.873 | 0.350 |  | 0.357 | 0.550 |  | 0.020 | 0.888 |
| City | 2484 | 1.00±3.41 |  |  | 0.59±1.93 |  |  | 0.39±1.72 |  |  |
| Rural | 661 | 0.86±3.33 |  |  | 0.54±1.91 |  |  | 0.38±1.66 |  |  |
| Current Residence |  |  | 9.247 | ﹤0.001 |  | 9.908 | ﹤0.001 |  |  | ﹤0.001 |
| Wuhan, Hubei | 560 | 1.54±3.34 |  |  | 0.83±1.89 |  |  | 0.63±1.66 |  |  |
| Other cities in Hubei | 389 | 0.56±3.67 |  |  | 0.31±2.03 |  |  | 0.20±1.86 |  |  |
| Other provinces and cities | 2191 | 0.89±3.34 |  |  | 0.56±1.90 |  |  | 0.36±1.69 |  |  |
| Overseas | 5 | 2.25±3.50 |  |  | 3.00±2.16 |  |  | 2.50±1.73 |  |  |
| Education |  |  | 3.816 | 0.004 |  | 2.844 | 0.023 |  | 4.518 | 0.001 |
| Elementary school or below | 7 | 1.14±1.68 |  |  | 0.57±1.27 |  |  | 0.57±0.79 |  |  |
| Middle school | 147 | 0.46±3.77 |  |  | 0.22±2.24 |  |  | 0.24±1.90 |  |  |
| High school | 467 | 0.52±3.66 |  |  | 0.48±2.19 |  |  | 0.13±1.89 |  |  |
| College | 2050 | 1.05±3.37 |  |  | 0.58±1.88 |  |  | 0.42±1.68 |  |  |
| Master degree and above | 474 | 1.21±3.11 |  |  | 0.78±1.70 |  |  | 0.58±1.57 |  |  |
| Marital status |  |  | 4.671 | 0.003 |  | 7.546 | ＜0.001 |  | 4.386 | 0.004 |
| Single | 832 | 1.31±3.19 |  |  | 0.83±1.85 |  |  | 0.57±1.63 |  |  |
| Married | 2145 | 0.83±3.46 |  |  | 0.47±1.93 |  |  | 0.33±1.72 |  |  |
| Seperated/divorced | 141 | 0.79±3.74 |  |  | 0.70±2.07 |  |  | 0.28±1.90 |  |  |
| Other | 27 | 1.85±2.09 |  |  | 0.96±1.56 |  |  | 0.56±1.31 |  |  |
| Occupation |  |  | 1.499 | 0.221 |  | 6.820 | 0.009 |  | 8.961 | 0.003 |
| Medical staff | 507 | 0.80±3.18 |  |  | 0.37±1.74 |  |  | 0.18±1.58 |  |  |
| Non-medical staff | 2638 | 1.00±3.44 |  |  | 0.62±1.95 |  |  | 0.43±1.73 |  |  |
| Monthly income (Yuan) |  |  | 1.688 | 0.150 |  | 1.174 | 0.320 |  | 1.133 | 0.339 |
| ＜2000 | 414 | 1.02±3.17 |  |  | 0.68±1.88 |  |  | 0.43±1.71 |  |  |
| 2000-5000 | 1040 | 0.93±3.62 |  |  | 0.52±2.04 |  |  | 0.33±1.80 |  |  |
| 5001-10000 | 947 | 1.11±3.14 |  |  | 0.62±1.82 |  |  | 0.45±1.56 |  |  |
| 10001-15000 | 386 | 1.02±3.36 |  |  | 0.64±1.83 |  |  | 0.45±1.69 |  |  |
| ＞15000 | 358 | 0.57±3.66 |  |  | 0.44±1.96 |  |  | 0.29±1.82 |  |  |
| Number of cohabitants |  |  | 1.163 | 0.322 |  | 5.830 | 0.001 |  | 1.841 | 0.138 |
| 0 | 50 | 1.80±2.73 |  |  | 1.18±1.80 |  |  | 0.78±1.35 |  |  |
| 1 | 239 | 0.92±3.55 |  |  | 0.99±2.11 |  |  | 0.54±1.89 |  |  |
| 2-3 | 1917 | 0.93±3.41 |  |  | 0.53±1.93 |  |  | 0.35±1.71 |  |  |
| ≥4 | 939 | 1.01±3.36 |  |  | 0.54±1.85 |  |  | 0.41±1.68 |  |  |
| Quarantine or not |  |  | 0.069 | 0.793 |  | 1.639 | 0.201 |  | 2.701 | 0.100 |
| Yes | 1436 | 0.98±3.58 |  |  | 0.62±2.00 |  |  | 0.45±1.81 |  |  |
| No | 1709 | 0.95±3.24 |  |  | 0.54±1.85 |  |  | 0.35±1.61 |  |  |
| Level of attention |  |  | 21.262 | ﹤0.001 |  | 16.943 | ﹤0.001 |  | 13.441 | ﹤0.001 |
| Significantly decrease | 54 | -1.67±5.51 |  |  | -0.56±2.66 |  |  | -0.70±2.52 |  |  |
| Decrease | 99 | -0.90±3.87 |  |  | -0.29±1.98 |  |  | -0.29±2.01 |  |  |
| Unchanged | 205 | 0.71±2.50 |  |  | 0.26±1.55 |  |  | 0.20±1.36 |  |  |
| Increase | 553 | 0.60±2.90 |  |  | 0.35±1.75 |  |  | 0.26±1.60 |  |  |
| Significantly increase | 2234 | 1.23±3.43 |  |  | 0.73±1.94 |  |  | 0.50±1.71 |  |  |
| Risk of infection |  |  | 33.407 | ﹤0.001 |  | 14.385 | ﹤0.001 |  | 12.218 | ﹤0.001 |
| Significantly decrease | 652 | 0.00±3.94 |  |  | 0.25±2.16 |  |  | 0.08±1.88 |  |  |
| Decrease | 489 | 0.42±3.49 |  |  | 0.36±1.91 |  |  | 0.23±1.76 |  |  |
| Unchanged | 715 | 0.99±2.95 |  |  | 0.62±1.69 |  |  | 0.44±1.53 |  |  |
| Increase | 737 | 1.38±2.76 |  |  | 0.62±1.69 |  |  | 0.48±1.48 |  |  |
| Significantly increase | 552 | 2.01±3.55 |  |  | 1.03±2.10 |  |  | 0.71±1.87 |  |  |
| Impact of daily life |  |  | 69.250 | ﹤0.001 |  | 48.641 | ﹤0.001 |  | 44.092 | ﹤0.001 |
| Significantly decrease | 35 | -3.11±5.10 |  |  | -1.06±2.62 |  |  | -1.29±2.59 |  |  |
| Decrease | 112 | -1.56±4.16 |  |  | -0.43±2.07 |  |  | -0.69±1.90 |  |  |
| Unchanged | 376 | -0.27±3.22 |  |  | 0.01±1.82 |  |  | -0.06±1.60 |  |  |
| Increase | 1204 | 0.72±3.00 |  |  | 0.36±1.80 |  |  | 0.27±1.54 |  |  |
| Significantly increase | 1418 | 1.80±3.32 |  |  | 1.03±1.89 |  |  | 0.73±1.73 |  |  |
| Self-perceived health status |  |  | 36.713 | ﹤0.001 |  | 8.465 | ﹤0.001 |  | 18.338 | ﹤0.001 |
| Fairly healthy | 1239 | 0.24±3.57 |  |  | 0.38±2.03 |  |  | 0.13±1.76 |  |  |
| Healthy | 1406 | 1.14±3.11 |  |  | 0.63±1.82 |  |  | 0.47±1.63 |  |  |
| General | 470 | 2.20±3.25 |  |  | 0.91±1.82 |  |  | 0.81±1.65 |  |  |
| Unhealthy but live independently | 27 | 3.70±2.89 |  |  | 1.37±2.06 |  |  | 1.22±1.74 |  |  |
| Unable to live independently | 3 | -0.67±9.87 |  |  | 0.67±5.86 |  |  | -1.67±3.79 |  |  |
| Mental health help-seeking |  |  | 31.566 | ﹤0.001 |  | 14.426 | ﹤0.001 |  | 19.454 | ﹤0.001 |
| Found and tried | 130 | -0.09±5.00 |  |  | 0.18±2.64 |  |  | 0.04±2.49 |  |  |
| Found but not tried | 45 | 0.13±3.45 |  |  | 0.27±2.25 |  |  | 0.07±1.88 |  |  |
| Not found yet | 80 | 2.39±3.99 |  |  | 1.30±2.20 |  |  | 1.18±1.97 |  |  |
| Not looked for | 1132 | 1.74±3.18 |  |  | 0.86±1.89 |  |  | 0.68±1.68 |  |  |
| No need to adjust | 1758 | 0.50±3.24 |  |  | 0.40±1.83 |  |  | 0.21±1.60 |  |  |
| Confirmed infected in personal network |  |  | 17.769 | ﹤0.001 |  | 4.514 | 0.034 |  | 7.961 | 0.005 |
| Yes | 472 | 1.57±3.40 |  |  | 0.75±1.97 |  |  | 0.60±1.74 |  |  |
| No | 2673 | 0.86±3.39 |  |  | 0.55±1.91 |  |  | 0.36±1.70 |  |  |

**Table S2. Factors affecting anxiety, depression, and stress in the 2^nd^ survey （n = 3814；X±S ;n/%）**

| Variable | N | Anxiety | t/F | P | Depression | t/F | P | Stress | t/F | P |
| --- | --- | --- | --- | --- | --- | --- | --- | --- | --- | --- |
| Gender |  |  | 0.012 | 0.913 |  | 0.004 | 0.950 |  | 0.030 | 0.864 |
| Male | 1312 | 0.01±2.51 |  |  | 0.00±2.53 |  |  | 0.01±1.88 |  |  |
| Female | 2502 | 0.00±2.40 |  |  | 0.00±2.44 |  |  | 0.00±1.80 |  |  |
| Age |  |  | 3.282 | 0.006 |  | 3.823 | 0.002 |  | 2.998 | 0.011 |
| ≤18 | 143 | -0.32±2.63 |  |  | -0.42±2.81 |  |  | -0.21±2.00 |  |  |
| 19-29 | 1009 | -0.02±2.54 |  |  | 0.09±2.63 |  |  | 0.04±1.90 |  |  |
| 30-39 | 1000 | 0.19±2.28 |  |  | 0.18±2.30 |  |  | 0.14±1.75 |  |  |
| 40-49 | 992 | -0.19±2.54 |  |  | -0.22±2.56 |  |  | -0.13±1.87 |  |  |
| 50-59 | 573 | 0.07±2.29 |  |  | 0.02±2.22 |  |  | -0.05±1.71 |  |  |
| ≥60 | 97 | 0.29±2.30 |  |  | 0.18±2.05 |  |  | 0.20±1.61 |  |  |
| Area |  |  | 48.897 | ﹤0.001 |  | 35.927 | ﹤0.001 |  | 31.045 | ﹤0.001 |
| City | 2963 | 0.15±2.34 |  |  | 0.13±2.36 |  |  | 0.09±1.76 |  |  |
| Rural | 851 | -0.51±2.69 |  |  | -0.44±2.78 |  |  | -0.31±2.01 |  |  |
| Current Residence |  |  | 17.233 | ﹤0.001 |  | 13.214 | ﹤0.001 |  | 21.570 | ﹤0.001 |
| Wuhan, Hubei | 1195 | 0.39±2.33 |  |  | 0.36±2.29 |  |  | 0.33±1.71 |  |  |
| Other cities in Hubei | 923 | -0.06±2.52 |  |  | -0.11±2.56 |  |  | -0.05±1.93 |  |  |
| Other provinces and cities | 1684 | -0.25±2.43 |  |  | -0.20±2.51 |  |  | -0.21±1.81 |  |  |
| Overseas | 12 | 0.97±2.60 |  |  | 0.94±2.50 |  |  | 0.51±2.07 |  |  |
| Education |  |  | 1.464 | 0.210 |  | 1.067 | 0.371 |  | 1.332 | 0.255 |
| Elementary school or below | 13 | 0.34±3.21 |  |  | 0.10±3.10 |  |  | 0.36±2.50 |  |  |
| Middle school | 117 | -0.06±2.48 |  |  | -0.14±2.39 |  |  | -0.12±1.90 |  |  |
| High school | 489 | -0.16±2.61 |  |  | -0.08±2.64 |  |  | -0.03±1.96 |  |  |
| College | 2418 | -0.02±2.46 |  |  | -0.03±2.50 |  |  | -0.03±1.84 |  |  |
| Master degree and above | 777 | 0.16±2.23 |  |  | 0.15±2.23 |  |  | 0.12±1.67 |  |  |
| Marital status |  |  | 3.766 | 0.010 |  | 1.969 | 0.116 |  | 2.964 | 0.031 |
| Single | 1104 | -0.03±2.52 |  |  | 0.05±2.64 |  |  | 0.01±1.91 |  |  |
| Married | 2537 | 0.00±2.41 |  |  | -0.03±2.39 |  |  | -0.01±1.78 |  |  |
| Separated/divorced | 150 | 0.46±2.19 |  |  | 0.29±2.32 |  |  | 0.24±1.82 |  |  |
| Other | 23 | -1.23±2.69 |  |  | -0.88±2.85 |  |  | -0.95±1.95 |  |  |
| Occupation |  |  | 0.395 | 0.530 |  | 0.275 | 0.600 |  | 0.023 | 0.879 |
| Medical staff | 1015 | 0.04±2.46 |  |  | -0.03±2.45 |  |  | 0.01±1.86 |  |  |
| Non-medical staff | 2799 | -0.01±2.43 |  |  | 0.01±2.47 |  |  | 0.00±1.81 |  |  |
| Monthly income (Yuan) |  |  | 3.730 | 0.005 |  | 3.007 | 0.017 |  | 2.175 | 0.069 |
| ＜2000 | 780 | -0.27±2.62 |  |  | -0.22±2.71 |  |  | -0.15±1.96 |  |  |
| 2000-5000 | 1145 | 0.08±2.49 |  |  | 0.04±2.48 |  |  | 0.02±1.88 |  |  |
| 5001-10000 | 1031 | -0.03±2.40 |  |  | -0.04±2.44 |  |  | 0.00±1.77 |  |  |
| 10001-15000 | 438 | -0.15±2.24 |  |  | 0.20±2.24 |  |  | 0.13±1.68 |  |  |
| ＞15000 | 420 | 0.19±2.16 |  |  | 0.18±2.21 |  |  | 0.08±1.68 |  |  |
| Number of cohabitants |  |  | 1.067 | 0.362 |  | 2.005 | 0.111 |  | 0.822 | 0.482 |
| 0 | 219 | 0.28±2.62 |  |  | 0.40±2.68 |  |  | 0.19±1.95 |  |  |
| 1 | 457 | 0.01±2.51 |  |  | -0.02±2.52 |  |  | -0.03±1.91 |  |  |
| 2-3 | 1926 | -0.02±2.41 |  |  | -0.03±2.44 |  |  | -0.01±1.80 |  |  |
| ≥4 | 1212 | -0.03±2.41 |  |  | -0.02±2.44 |  |  | -0.01±1.82 |  |  |
| Quarantine or not |  |  | 1.010 | 0.315 |  | 1.316 | 0.243 |  | 3.018 | 0.082 |
| Yes | 432 | 0.11±2.45 |  |  | 0.13±2.54 |  |  | 0.14±1.86 |  |  |
| No | 3382 | -0.01±2.44 |  |  | -0.02±2.46 |  |  | -0.02±1.82 |  |  |
| Level of attention |  |  | 16.410 | ﹤0.001 |  | 11.319 | ﹤0.001 |  | 19.673 | ﹤0.001 |
| Reduced a lot | 393 | -0.73±2.78 |  |  | -0.62±2.91 |  |  | -0.60±2.07 |  |  |
| Reduced a bit | 1115 | -0.16±2.27 |  |  | -0.05±2.36 |  |  | -0.14±1.69 |  |  |
| No significant changes | 1152 | 0.34±2.16 |  |  | 0.30±2.18 |  |  | 0.26±1.59 |  |  |
| Increased a bit | 398 | 0.21±2.37 |  |  | 0.12±2.34 |  |  | 0.20±1.78 |  |  |
| Increased a lot | 756 | -0.01±2.79 |  |  | -0.12±2.77 |  |  | 0.01±2.12 |  |  |
| Risk of infection |  |  | 96.363 | ﹤0.001 |  | 75.429 | ﹤0.001 |  | 109.723 | ﹤0.001 |
| Reduced a lot | 1586 | -0.73±2.70 |  |  | -0.68±2.75 |  |  | -0.57±1.98 |  |  |
| Reduced a bit | 1156 | 0.04±2.16 |  |  | 0.08±2.23 |  |  | 0.04±1.59 |  |  |
| No significant changes | 853 | 0.91±1.80 |  |  | 0.84±1.83 |  |  | 0.71±1.42 |  |  |
| Increased a bit | 174 | 1.33±1.95 |  |  | 0.95±1.89 |  |  | 1.01±1.50 |  |  |
| Increased a lot | 45 | 2.22±2.29 |  |  | 2.16±2.34 |  |  | 2.01±1.89 |  |  |
| Impact of daily life |  |  | 77.996 | ﹤0.001 |  | 79.171 | ﹤0.001 |  | 79.886 | ﹤0.001 |
| Reduced a lot | 289 | -1.67±2.96 |  |  | -1.70±3.01 |  |  | -1.21±2.10 |  |  |
| Reduced a bit | 586 | -0.82±2.63 |  |  | -0.83±2.73 |  |  | -0.60±1.90 |  |  |
| No significant changes | 1708 | 0.12±2.11 |  |  | 0.13±2.15 |  |  | 0.06±1.58 |  |  |
| Increased a bit | 848 | 0.52±2.15 |  |  | 0.50±2.12 |  |  | 0.36±1.65 |  |  |
| Increased a lot | 383 | 0.82±2.73 |  |  | 0.88±2.69 |  |  | 0.77±2.15 |  |  |
| Self-perceived health status |  |  | 53.892 | ﹤0.001 |  | 43.184 | ﹤0.001 |  | 48.974 | ﹤0.001 |
| Fairly healthy | 1992 | -0.49±2.56 |  |  | -0.44±2.61 |  |  | -0.34±1.89 |  |  |
| Healthy | 1525 | 0.39±2.13 |  |  | 0.34±2.17 |  |  | 0.26±1.64 |  |  |
| General | 275 | 1.23±2.19 |  |  | 1.12±2.17 |  |  | 0.93±1.71 |  |  |
| Unhealthy but live independently | 20 | 2.15±2.78 |  |  | 2.27±2.66 |  |  | 1.41±1.80 |  |  |
| Unable to live independently | 2 | -0.16±7.63 |  |  | -1.15±6.77 |  |  | -1.09±4.10 |  |  |
| Mental health help-seeking |  |  | 35.054 | ﹤0.001 |  | 39.559 | ﹤0.001 |  | 37.568 | ﹤0.001 |
| Found and tried | 264 | -0.60±2.69 |  |  | -0.66±2.83 |  |  | -0.45±1.92 |  |  |
| Found but not tried | 79 | 0.03±2.60 |  |  | 0.54±2.37 |  |  | 0.05±1.71 |  |  |
| Not found yet | 82 | 1.35±2.37 |  |  | 1.31±2.45 |  |  | 1.05±1.94 |  |  |
| Not looked for | 1174 | 0.57±2.31 |  |  | 0.61±2.31 |  |  | 0.44±1.78 |  |  |
| No need to adjust | 2215 | -0.28±2.40 |  |  | -0.31±2.42 |  |  | -0.22±1.77 |  |  |
| Confirmed infected in personal network |  |  | 34.165 | ﹤0.001 |  | 32.741 | ﹤0.001 |  | 37.665 | ﹤0.001 |
| Yes | 720 | 0.48±2.33 |  |  | 0.47±2.82 |  |  | 0.37±1.76 |  |  |
| No | 3094 | -0.11±2.45 |  |  | -0.11±2.50 |  |  | -0.09±1.83 |  |  |
